# Application of propensity scores to estimate the association between government subsidy and injection use in primary health care institutions in China

**DOI:** 10.1186/1472-6963-13-183

**Published:** 2013-05-21

**Authors:** Yuqing Tang, Xiaopeng Zhang, Chunyan Yang, Lianping Yang, Hongtao Wang, Xinping Zhang

**Affiliations:** 1 School of Medicine and Health Management, Tongji Medical College, HuaZhong University of Science and Technology, Wuhan, HuBei Province, China

**Keywords:** Propensity score, Primary health care, Government subsidy, Injection use, China

## Abstract

**Background:**

The problem posed by therapeutic injection is a clinical practice issue that influences health care quality and patient safety. Although sufficient government subsidy was one of the 12 key interventions to promote rational drug use initiated by WHO (World Health Organization), limited information is available about the association between government subsidy and injection use in primary health care institutions. In 2009, National Essential Medicines System (NEMS) was implemented in China. The subsidy policy plays an important role in maintaining primary health care institutions. This study explores the impact of government subsidies on the injection use in primary health care institutions in China.

**Methods:**

126 primary health institutions were included in this study. Institutions were divided into two groups (intervention and control groups) according to the median GS (General subsidy per personnel). Propensity score matching (PSM) was used to minimize the observed covariate differences in the characteristics of the primary institutions between the two groups. Kappa score was calculated to determine the consistency between the groups. Paired chi-square test and Relative Risk (RR) were calculated to compare the differences in injection use between the groups.

**Results:**

Among all the investigated prescriptions, the overall percent of people who received an injection prescribed was 36.96% (n = 12600). PSM showed no significant covariate difference among the 34 groups obtained through this analysis. Kappa score (k = −0.082, p = 0.558) indicated an inconsistency between groups and paired chi-square test revealed a significant difference (*p* < 0.05) in injection use between the two groups. Relative Risk = 0.679 (95%CI [0.485, 0.950]) indicate that high General subsidy per personnel is a protective factor for primary health care institutions to prescribe injections properly. The intervention group obtained a higher possibility of using injection properly.

**Conclusions:**

The overall effect of government subsidy on the use of injection was positively significant. However, the mechanism by which government subsidy influence injection administration remains unclear, and thus requires further study.

## Background

The quality improvement of medical service and patient safety is of great importance. The focus on patient safety has intensified since 1999 [1], and health care quality improvement has gained increasing attention. Members of international and national organizations, such as the Institute for Healthcare Improvement, WHO, the National Quality Forum, and the Agency for Healthcare Research and Quality have postulated theories, developed guidelines, and published reports on this area. However, theoretical concepts have not been easily or completely translated into clinical practice regardless of the increasing academic activities [2].

The problem posed by therapeutic injection is a clinical practice issue that influences both health care quality and patient safety. WHO estimates that approximately 16 billion injections are used annually in developing and transitional countries. In particular, the ratio of vaccination injection to therapeutic injections given is 0.5:9.5 [3]. Since most medications used in primary health care institutions can be taken orally, this ratio, along with population-based injection frequency surveys, has indicated the overuse of therapeutic injections in developing and transitional nations. This is a severe problem in China. A large prescription sample in urban community health care institutions across China has shown that the percentages of injection prescription were 35.4%, 32.4%, and 33.9% in 2007, 2008, and 2009, respectively [4]. Overuse of injections results in serious consequences.

Multiple factors other than clinical considerations can influence the decision to prescribe injections. Considering the characteristics of individual patients and doctors, patient characteristics, such as age, lower socio-economic status, and higher co-morbidity have significant effects on prescription behaviour [5-7]. Physician characteristics, including gender, age, time since graduation, and duration of practice, significantly influence the prescription behaviour [5,8,9]. Considering the characteristics of the facilities and social activities, evidence has indicated that organizational culture may also be an important factor in health care performance [10]. Exposure to direct information from pharmaceutical companies has been associated with higher prescription frequency, higher costs, or lower prescription quality [11]. Specific geographical demand and supply factors of the areas are also related to the patterns of injection prescriptions [12].

China has experienced significant changes in social and economic structures since the onset of economic reforms in 1978. In the healthcare sector these changes have led to a decreased reliance on state funds [13], decentralization of public health services, increased autonomy of health facilities, and increased freedom in health worker movements. An association between economic reforms and the growing inequality in health care service access, increasing medical care cost, decreasing public finances, and increasing funds by user fees has been observed [14]. Hospitals in China provide subsidized clinical services through the fee-for-service system allowing physicians to add a 15% mark-up on medication. This has an influence on physician prescribing factors. Compared with oral formulations, injections are more expensive. The higher cost of injections entails more profit for the physician [15]. Studies have indicated the mark-up of injections can reach >15% [16].

In 2009, China launched the essential medicines system. This system includes a list of drug formulations, production, supply, procurement, use, pricing, payment, reimbursement, quality control, monitoring, and evaluation for the purpose of increasing medicine availability, reducing drug safety hazards, and minimizing irrational drug prescription. All of the primary health care institutions (excluding hospitals and private providers) in urban and rural areas must acquire and use essential medicines. Government-owned public primary care providers must sell essential medicines at 0% mark-up in accordance with current laws [17]. Since 1997, the National Development and Reform Commission adjusted pharmaceutical prices for 24 times. However, regulated price markdowns have had a limited impact on the increase in health expenses. Compared with the multiple price adjustments, the essential medicines system is one of the more effective policies for reducing the soaring drug costs [18]. To support the operation of primary health care institutions and defray the aforementioned costs, the government has set up a subsidy scheme [19]. Based on this scheme, the government is responsible for public health service, emergency expenditures, and personnel expenses, which include funding for retired personnel, staff training, and recruitment. If all of these subsidy schemes fail to cover the health expenses, then the balance from the revenue and total costs become the amount of the subsidy. In this kind of scheme, the income of primary health care providers comes mainly from the medical service charges and government subsidies.

Government subsidies can significantly decrease the drug expenses of a facility, thereby decreasing the use of injection [20]. However, few statistical data on the direct correlation between injection prescription and subsidies have been reported, although WHO has claimed that sufficient government subsidy is needed to promote rational drug use. This particular area of study has gained attention, but “robust” experimental study designs are limited [21]. This study aims to fill the gap by examining a sample of primary health care institutions across China based on the essential medicine system to provide evidence-based recommendations for policy development in primary healthcare systems in China.

## Methods

### Data source and sampling

Using a uniform questionnaire, data were obtained from the openly available survey of the National Essential Drug System Monitoring Project performed by the Ministry of Health in six provinces in China from October to December 2011.

The following sampling facilities were used: Shandong and Liaoning in the east of China, Hubei and Shanxi in the central part of China, and Shaanxi and Sichuan in the western part of China. Investigations included 126 primary health care institutions (21 for each province). These primary health care institutions gave a representative sample for the overall development of primary health care services in China.

A systematic method for prescription sampling using data collected from January to September 2011 yielded 100 randomly sampled prescriptions (10 to 15 prescriptions per month) for each facility.

### Statistical variables

We used a matched pair design with propensity score matching (PSM) to analyze the correlation between government subsidies and injection use. Since there is no standard to decide whether a primary health institutions has a high or low government subsidy, General subsidy per personnel (GS) was used as a criterion. The two most important components of the current subsidy scheme are public health service and emergency expenditure, as well as personnel expenses (funding for retired personnel, staff training, and recruitment). Approved allocation for public health expenditure was comprehensively measured by the amount, which relies heavily on the approved number of personnel; quality and unit cost, approved allocation for personnel expenses was considered by the approved number of personnel and their salary [22]. Since the number of personnel is a key consideration in the subsidy scheme, compared to the total government subsidy, GS more accurately reflects the standard of government subsidy for this study.

GS is calculated as follows:

$$\mathrm{General subsidy per personnel}=\frac{\mathrm{government subsidy}}{\mathrm{The number of staff}}$$

The facilities were grouped according to the median GS. The facilities with a GS higher than the median were defined as the intervention group and those with lower GS than the median were defined as the control group with reference to some similar study [23].

The outcome indicator (i.e., the injection use) was determined at a facility level. According to an international standard recommended by WHO, the facilities with injection use rate < 24.1% were defined as ‘rational’ and facilities with an injection rate >24.1% were defined as ‘otherwise’ [4].

### PSM

Propensity score, first proposed by Rosenbaum and Rubin in 1983 [24], is a function related to multiple covariates and represents the combined result of all covariates. PSM balances the covariate difference between intervention and control groups. The propensity score also provides a similar randomized processing method to non-randomized observational studies [25].

PSM estimators can evaluate the effects of intervention by comparing the outcomes in the intervention group with the same results in the control group. In this case, intervention represents the high GS and the outcomes refer to the difference in the use of injection between the intervention group and the control group.

Control group facilities are suitable matches for intervention group facilities if they have similar observed characteristics as evaluated by a particular distance metric, i.e., the propensity score from a logistic regression model in this case.

PSM was implemented in two steps: (1) the conditional probability of being highly compensated (i.e., the propensity score) was calculated; and (2) the nearest neighbors in close proximity to the exposed facility propensity scores are selected from the control group.

There is an ongoing controversy in the literature as to which variables to include in the propensity score model. Variables that are unrelated to the exposure but related to the outcome should always be included in a PS model [26]. As to the confounders that associated with both exposure and outcome, evidence has indicted the omitting of such variables from the propensity score model would result in biased estimation of the intervention effect [27]. So in this research, we have selected all variables available that are potentially related with the inject prescription. The covariates incorporated in the logistic regression model for PSM include the following: the province and category of the institutions; the number of staff, outpatients, and emergency patients; medical, examination, and drug income; and the average salary of the staff. Since we take GS as a criterion for government subsidy in lieu of total government subsidy, institutions that have the ability to obtain higher subsidy (given the same stuff) or smaller number of staff (given the same subsidy) are more likely to be in the intervention group. Table 1 summarizes the description of these variables. In this paper, nearest neighbor matching was performed using a caliper value of 0.003 [28]. We used the caliper matching with one-to-one matches based on the propensity score.

**Table 1:** Variables descriptions in the logistic model of PSM

	**Variable name**	**Description**
Independent variable	GS	Intervention group, control group
Dependent variable	The number of staff	Continuous
	the number of outpatients and emergency patients	Continuous
	medical income	Continuous
	examination income	Continuous
	drug income	Continuous
	the average salary of the staff	Continuous
	the province of institutions	Hubei, Liaoning, Shandong, Shanxi, Shaanxi, Sichuan
	the category of institutions	Community Health Center (station), Township primary health institutions

### Data analysis

To analyze the use of injection between groups, the injection rate < 24.1% was defined rational injection use and the institutions were evaluated as “1;” or otherwise, “0.” Independent *t*-test or chi-square test to analyze the difference between intervention and control groups before and after PSM was performed. For the categorical variables (province and category of institutions), chi-square test was used to analyze the difference between groups. For continuous variables (number of staff, outpatients, and emergency patients; medical, examination, drug income; and average salary of the staff), independent *t*-test was used. The rational use of injection was the outcome indicator that we analyzed. After PSM, kappa score was calculated to determine the consistency between the groups and paired chi-square test to determine the difference between intervention and control groups. Relative Risk (RR) was calculated to estimate the association between government subsidy and injection use. The injection use rate and chi-square test was used to analyze the difference between groups as a further indicator of rate change between the groups. Statistical significance was accepted at *p*-value ≤ 0.05 (SPSS 12.0).

## Results

### Characteristics of the participating primary health care institutions

Our data showed that the use of injections in primary health care institutions does not meet the standards. Only 43 out of 126 institutions satisfied the criterion, which accounts for 34.13% of the total institutions. Among all the investigated prescriptions the overall percent with an injection prescribed was 36.96% (n = 12600) (for rational injection use institutions and irrational injection use institutions, this rate was 14.26% and 48.73% respectively). About two-thirds of the primary institutions are located in the rural areas. Other descriptive statistics for the variables are presented in Table 2.

**Table 2:** Injection use and other characteristics of investigated primary health institutions

**Variables**	**Mean ± SD/frequency(Percent)**
Injection use	
Rational	43(34.13)
Irrational	83(65.87)
Province	
Hubei	21(16.67)
Liaoning	21(16.67)
Shandong	21(16.67)
Shanxi	21(16.67)
Shaanxi	21(16.67)
Sichuan	21(16.67)
Category of institutions	
Community Health Center(station)	35(27.78%)
Township primary health institutions	91(72.22%)
Number of staff	50.17 ± 37.96
Number of outpatients and emergency patients	21885.42 ± 21955.53
Medical income (ten thousands Yuan)	643.00 ± 128.45
Examination income (ten thousands Yuan)	299.00 ± 22.94
Drug income (ten thousands Yuan)	985.83 ± 96.03
Average salary of the staff (Yuan)	2368.53 ± 683.51

Note: the total number of sampled institutions was 126. For continuous and categorical variables, mean ± standard deviation and Frequency (Percent) was used, respectively.

### Propensity score estimation

The propensity score was estimated using a logistic regression model of the probability of receiving a high government subsidy based on the following: number of outpatients and emergency patients; medical, examination, and drug income; average salary of the staff; the number of staff; as well as province and category of institutions. We obtained 34 pairs after PSM. The utilization of the total sample was 54% (68 out of 126). Table 3 shows the covariate in the pre- and post-match samples for the intervention and control groups. The medical income and the average salary of the staff exhibited a significant difference between the groups (*p* < 0.05) before PSM. After PSM, the differences were insignificant (*p* > 0.05). Three variables obtained a higher *p*-value after PSM. Thus, PSM resulted in a more balanced distribution of the variables between control and intervention groups. By contrast, three variables resulted in a slightly lower *p*-value after PSM.

**Table 3:** Variables for per- and post-matched samples for the intervention and comparison group

	**Pre-PSM approach**			**Post-PSM approach**		
	**Control group**	**Intervention group**	**P**	**Control group**	**Intervention group**	**P**
Province						
Hubei	14(22.22)	7(11.11)	0.06	5(14.70)	5(14.70)	0.96^a^
Liaoning	10(15.87)	11(17.46)		5(14.70)	4(11.80)	
Shandong	8(12.70)	13(20.63)		7(20.60)	6(17.60)	
Shanxi	13(20.63)	8(12.70)		3(8.80)	6(17.60)	
Shaanxi	5(7.94)	16(25.40)		4(11.80)	4(11.80)	
Sichuan	11(17.46)	10(15.87)		10(29.40)	9(26.50)	
Category of institutions						
Community Health Center (station)	18(28.60)	17(27.00)	0.84	8(23.50)	7(20.60)	0.77
Township primary health institutions	45(71.40)	46(73.00)		26(76.50)	27(79.40)	
The number of staff	56.37 ± 45.07	43.98 ± 28.21	0.07	51.15 ± 38.05	41.24 ± 28.68	0.23
The number of outpatients and emergency patients	22731.16 ± 18330.00	21039.6 ± 25185.96	0.67	23201.76 ± 20435.07	19654.26 ± 17183.63	0.44
The medical income (ten thousands Yuan)	156.62 ± 156.64	100.2 ± 115.21	0.02	142.16 ± 147.86	122.01 ± 110.25	0.53
The examination income (ten thousands Yuan)	24.88 ± 37.90	21.00 ± 40.79	0.58	34.43 ± 48.36	20.32 ± 25.21	0.14
The drug income (ten thousands Yuan)	99.69 ± 74.40	92.38 ± 166.95	0.73	101.451 ± 83.69	67.14 ± 57.57	0.053
The average salary of the staff (Yuan)	2117.37 ± 690.21	2619.69 ± 580.78	0.00	2486.08 ± 639.97	2498.22 ± 533.89	0.93

Note: for continuous and categorical variables, mean ± standard deviation and Frequency (Percent) was used respectively. Differences between groups were tested by independent *t* test for continuous variables, chi-square test for categorical variables. All p-values were two tailed. ”a” stands for Fisher’s exact probability for expected count less than 5.

### Effect of government subsidy on injection rate

The McNemar test showed that the rational use of injection between intervention and control groups was significantly different (*p* < 0.05). Furthermore, Kappa score, which indicates an inconsistency between the two groups (*k* = −0.082, *p* = 0.558), was calculated. RR score (RR = 0.679, 95%CI [0.485, 0.950]) indicate that GS is a protective factor for primary health care institutions to prescribe injections properly. Compared to the control group, the intervention group exhibited a lower possibility (67.9% the possibility in control group) of improper injection use. The percent of prescription with an injection prescribed was 42.12% (n = 3400) and 33.79% (n = 3400) for control group and intervention group respectively. The difference was statistically significant (χ^2^ = 50.01, P < 0.0001). These results show that government subsidy influences injection use.

## Discussion

Overuse of injections is still a severe problem in china as compared to an international standard (13.4%-24.1%) recommended by WHO. The overall percent with an injection prescribed was 36.96% (n = 12600) in this study. This indicates that at least 3 out of 10 patients received prescriptions for injections in primary health care institutions in china. Since evidence has proven that injection prescriptions account for more than one third of prescription before 2009 (35.4% for 2007 and 33.9% for 2008) [4], there seems to be no significant drop in the number of injection prescriptions. This conclusion is in accordance with a previous study which shows that the NEMP interventions failed to fulfil its goal in reducing irrational use of injections [29].

By using a survey data set from China, this study demonstrated that government subsidy has a significant effect. High government subsidy may increase the possibility of rational injection prescription. Few studies have used representative survey data to indicate the effect of government subsidies on injection prescription utilization. A previous study has suggested that irrational injection prescription use is observed in primary health care institutions in rural areas because of the insufficient government subsidy for both the facilities and the staff [20]. Another study examined the prescription behavior of village doctors and reported that government subsidy can help in the improvement of prescription quality and reduce the use of injections and antibiotics [30]. However, these studies were naturalistic and no statistical evidence corroborated the conclusions presented.

Our results show significant implications from a policy perspective. Despite the Policymakers’ continuous search for evidence, only a small number of regulatory measure evaluations have been conducted [31]. Based on the results obtained in this study, government subsidy has a significant effect on injection prescribing factors. High government subsidy may increase the possibility of rational injection prescriptions based on our survey data set from China. However, the mechanism by which government subsidy affects injection prescriptions remains unclear, and thus requires further studies. Given that the Chinese government increases the financial investment in health sectors annually, determining the link between government subsidy and rational drug use is necessary for proper budgetary planning.

In addition to the positive relation (high GS relate with high rational injection use), there is still negative association (high GS with low rational injection use) presented in Table 4. One likely explanation is that there are some other complex issues. This might include staff educational level and idiosyncratic beliefs on the part of the patient. These variables are not included in our study. Further investigation in this area may explore the limitations of the data collection methods used.

**Table 4:** Case summary after PSM-matching

		**Control group**		**Total**
		**Rational use**	**Irrational use**	
Intervention group	Rational use	2	13	15
	Irrational use	4	15	19
Total		6	28	34

Note: the numbers in every cell stands for pairs under the given condition.

From a methodological perspective, our analysis used PSM to determine the effect of government subsidy on drug use. This study is the first to report on the relationship between government subsidy and drug use in China. To avoid the risk of bias, we used propensity scores to estimate the probability that a health sector would receive a high government subsidy based on specific characteristics. The propensity score was estimated using logistic regression, with the subsidy as the outcome and the background covariates as the predictive variables. The covariates in both groups within the propensity-score strata were uniformly distributed. In our study, 34 pairs of health sectors shared close propensity scores and similar background covariates. The uniformity in the measured risk factor distribution indicates that the distribution of unmeasured variables is balanced, although propensity scores cannot remove hidden biases except to the extent that the unmeasured prognostic variables are correlated with the measured covariates used to compute the score. Therefore, efficient development of healthcare policies can be supported using propensity score analysis.

Although this study has explored the positive effects of government subsidies may have on injection prescriptions, our study has some data limitations which made the conclusions of this study should be interpreted with caution. Annual performance evaluations rather than a sample of a one-year cross-section would have been more informative. Furthermore, the evaluation method only allowed us to provide a causal inference on the effect of a high government subsidy on injection prescriptions to a limited extent. Confounding factors such as staff educational level and patient beliefs were not included in this study. Despite these limitations, our study has provided information for further investigations in this field.

## Conclusions

This study has observed the injection prescribing practices in primary health care institutions in China and the positive effects of government subsidies may have on injection use. The sample consists of a representative sample of primary health care institutions in six provinces. PSM, which may partially adjust the confounders, was used in our study to avoid possible bias that may influence the results. Our findings suggested that a high government subsidy may promote a rational approach towards injection prescription practices. However, we did not investigate the mechanism by which government subsidy affects injection prescriptions. Thus, further study on the relationship between injection prescription and injection use is recommended.

## Abbreviations

WHO: World Health Organization; NEMS: National Essential Medicines System; GS: General subsidy per personnel.

## Competing interests

The authors declare that they have no competing interest.

## Authors’ contributions

Study concept and design: Xinping Z and YT. Data Collection: HW, LY and CY. Statistical analysis: Xiaopeng Z, HW and LY. Analysis and interpretation of data: YT, Xiaopeng Z. Draft of the manuscript: YT and Xinping Z. Critical revision of the manuscript for important intellectual content: Xinping Z, YT. Study supervision: Xinping Z. All authors read and approved the final manuscript.

## Pre-publication history

The pre-publication history for this paper can be accessed here:

http://www.biomedcentral.com/1472-6963/13/183/prepub

## References

[B1] Wachter R Patient safety at 10 years: how far have we come? What’s next? OR Manager 2010 3 1 5 7 20387299

[B2] Dickey NW Corrigan JM Denham CR Ten-year retrospective review J Patient Safe 2010 6 1 1 4 10.1097/PTS.0b013e3181d321ca 22130296

[B3] Organization WH Safety of injections: Global facts and figures 2004 Geneva: World Health Organization

[B4] Li Y Xu J Wang F Wang B Liu L Hou W Fan H Tong Y Zhang J Lu Z Overprescribing in China, driven by financial incentives, results in very high use of antibiotics, injections, and corticosteroids Health Aff (Millwood) 2012 31 5 1075 1082 10.1377/hlthaff.2010.0965 22566449

[B5] Huang N Chou YJ Chang HJ Ho M Morlock L Antibiotic prescribing by ambulatory care physicians for adults with nasopharyngitis, URIs, and acute bronchitis in Taiwan: a multi-level modeling approach Fam Pract 2005 22 2 160 167 10.1093/fampra/cmh734 15722399

[B6] Akkerman AE van der Wouden JC Kuyvenhoven MM Dieleman JP Verheij TJ Antibiotic prescribing for respiratory tract infections in Dutch primary care in relation to patient age and clinical entities J Antimicrob Chemother 2004 54 6 1116 1121 10.1093/jac/dkh480 15546973

[B7] Majeed A Moser K Age- and sex-specific antibiotic prescribing patterns in general practice in England and Wales in 1996 British J Gen Pract J Royal College Gen Pract 1999 49 446 735 736 PMC1313505 10756619

[B8] Cadieux GTR Dauphinee D Libman M Predictors of inappropriate antibiotic prescribing among primary care physicians CMAJ 2007 177 877 883 10.1503/cmaj.070151 17923655 PMC1995156

[B9] Kozyrskyj AL Dahl ME Chateau DG Mazowita GB Klassen TP Law BJ Evidence-based prescribing of antibiotics for children: role of socioeconomic status and physician characteristics CMAJ 2004 171 2 139 145 10.1503/cmaj.1031629 15262882 PMC450362

[B10] Scott T Mannion R Marshall M Davies H Does organisational culture influence health care performance? A review of the evidence J Health Serv Res Policy 2003 8 2 105 117 10.1258/135581903321466085 12820673

[B11] Spurling GK Mansfield PR Montgomery BD Lexchin J Doust J Othman N Vitry AI Information from pharmaceutical companies and the quality, quantity, and cost of physicians’ prescribing: a systematic review PLoS Med 2010 7 10 e1000352 10.1371/journal.pmed.1000352 20976098 PMC2957394

[B12] Choi KH Park SM Lee JH Kwon S Factors affecting the prescribing patterns of antibiotics and injections J Korean Med Sci 2012 27 2 120 127 10.3346/jkms.2012.27.2.120 22323857 PMC3271283

[B13] Hsiao WC The political economy of Chinese health reform Health Econ Policy Law 2007 2 Pt 3 241 249 18634648 10.1017/S1744133107004197

[B14] Yu X Li C Shi Y Yu M Pharmaceutical supply chain in China: current issues and implications for health system reform Health Policy 2010 97 1 8 15 10.1016/j.healthpol.2010.02.010 20307912

[B15] Injection safetyhttp://www.who.int/mediacentre/factsheets/fs231/en/

[B16] Ai-tian Y Xin-tai L Study on the impact of essential medicine system on Out-patient service in township hospitals of Shandong province Chinese Health Econ 2011 04 20 22

[B17] The State Council of China Implementation plan for the recent priorities of the health care system reform (2009–2011) 2009 Beijing: The State Council of China

[B18] Guan X Liang H Xue Y Shi L An analysis of China’s national essential medicines policy J Public Health Policy 2011 32 3 305 319 10.1057/jphp.2011.34 21614030

[B19] The State Council of China Opinions on establishing and implementation of compensation mechanism of the primary health care institutions 2010 Beijing: The State Council of China

[B20] Xinping Z Xianji W Research on the reasons of irrational Use of medicines Chinese Primary Health Care 2005 12 23 26

[B21] Gosden T Forland F Kristiansen IS Sutton M Leese B Giuffrida A Sergison M Pedersen L Capitation, salary, fee-for-service and mixed systems of payment: effects on the behaviour of primary care physicians Cochrane Database Syst Rev 2000 3 CD002215 10.1002/14651858.CD002215 PMC9879313 10908531

[B22] Wang Xiao-rong P-X Implementation of the compensation mechanism of essential drug system in primary health care institutions Chinese Health Econ 2011 30 9 27 28

[B23] Xu J Wang W Li Y Zhang J Pavlova M Liu H Yin P Lu Z Analysis of factors influencing the outpatient workload at Chinese health centres BMC Heal Serv Res 2010 10 151 10.1186/1472-6963-10-151 PMC2904333 20525381

[B24] Rosenbaum P Rubin D The central role of the propensity score in observational studies for causal effects Biometrika 1983 70 41 55 10.1093/biomet/70.1.41

[B25] Farzadfar F Murray CJ Gakidou E Bossert T Namdaritabar H Alikhani S Moradi G Delavari A Jamshidi H Ezzati M Effectiveness of diabetes and hypertension management by rural primary health-care workers (Behvarz workers) in Iran: a nationally representative observational study Lancet 2012 379 9810 47 54 10.1016/S0140-6736(11)61349-4 22169105

[B26] Brookhart MA Schneeweiss S Rothman KJ Glynn RJ Avorn J Sturmer T Variable selection for propensity score models Am J Epidemiol 2006 163 12 1149 1156 10.1093/aje/kwj149 16624967 PMC1513192

[B27] Austin PC Grootendorst P Anderson GM A comparison of the ability of different propensity score models to balance measured variables between treated and untreated subjects: a Monte Carlo study Stat Med 2007 26 4 734 753 10.1002/sim.2580 16708349

[B28] Austin PC Some methods of propensity score matching had superior performance to others:results of an empirical investigation and Monte Carlo simulations Biomet J 2009 51 171 184 10.1002/bimj.200810488 19197955

[B29] Yang L Liu C Ferrier JA Zhou W Zhang X The impact of the National Essential Medicines Policy on prescribing behaviours in primary care facilities in Hubei province of China Health Policy Plan 2012 [Epub ahead of print] 10.1093/heapol/czs116 23161585

[B30] Dong L Yan H Wang D Drug prescribing indicators in village health clinics across 10 provinces of Western China Fam Pract 2011 28 1 63 67 10.1093/fampra/cmq077 20876222

[B31] Sturm H Austvoll-Dahlgren A Aaserud M Oxman AD Ramsay C Vernby A Kosters JP Pharmaceutical policies: effects of financial incentives for prescribers Cochrane Database Syst Rev 2007 3 CD006731 10.1002/14651858.CD006731 17636851

